# Diversity of heart failure phenotypes in transthyretin amyloid cardiomyopathy. More than just heart failure with preserved ejection fraction

**DOI:** 10.1080/07853890.2024.2418965

**Published:** 2024-10-26

**Authors:** Anouk Achten, Steven A. Muller, Sandra Sanders-van Wijk, Manon G. van der Meer, Pim van der Harst, Peter van Tintelen, Anneline SJM te Riele, Vanessa van Empel, Marish IFJ Oerlemans, Christian Knackstedt

**Affiliations:** a Department of Cardiology, Maastricht University, Cardiovascular Research Institute Maastricht (CARIM), Maastricht, the Netherlands; b Department of Cardiology, Zuyderland Medical Center, the Netherlands; c Department of Cardiology, University Medical Center Utrecht, the Netherlands; d Netherlands Heart Institute, Utrecht, the Netherlands; e Member of the European Reference Network for rare, low prevalence and complex diseases of the heart: ERN GUARD-Heart, (ERN GUARDHEART; http://guardheart.ern-net.eu);; f Department of Genetics, University Medical Center Utrecht, Utrecht University, Utrecht, the Netherlands

**Keywords:** Transthyretin cardiac amyloidosis, Echocardiography, diagnostic guidelines, heart failure, left ventricular dilatation

## Abstract

**Introduction:**

Current guidelines recommend suspecting transthyretin amyloid cardiomyopathy (ATTR-CM) in patients over 65 years of age with unexplained left ventricular (LV) hypertrophy in a non-dilated LV, heart failure (HF) and preserved ejection fraction (HFpEF), hypertrophic cardiomyopathy or severe aortic stenosis. However, there is evidence indicating a high prevalence of ATTR-CM in other HF phenotypes. As such, this study aimed to characterize the diversity of HF phenotypes of ATTR-CM by examining the LV ejection fraction and LV dilatation using echocardiography.

**Methods:**

This multicentre, retrospective observational study included patients diagnosed with ATTR-CM between 2015–2023. The diagnosis was based on a positive cardiac biopsy or positive bone scintigraphy without monoclonal gammopathy. Echocardiographic measurements were categorized according to LV ejection fraction (LVEF) into HFpEF (LVEF ≥50%), HF with mildly reduced EF (HFmrEF, LVEF 40–49%), and HF with reduced EF (HFrEF, LVEF <40%). LV cavity size was categorized by LV end-diastolic diameter (LVEDD) and volume index (LVEDVi) as normal, moderately increased and severe dilatation.

**Results:**

The study included 135 patients with ATTR-CM (mean age, 78 years; 89% male; 89% wild-type ATTR-CM). Most patients were screened for ATTR-CM because of unexplained HF and increased LV wall thickness (57%). Echocardiography showed LVEF <50% in 60% of the patients, with a significant portion presenting with HFrEF. Patients with LVEF <50% had higher NYHA class and elevated N-terminal pro-B-type natriuretic peptide levels than HFpEF patients. LV dilatation was observed in 43% of the patients, with 10% presenting with both LVEF <50% and severe LV dilatation.

**Conclusion:**

This study revealed significant variability in HF phenotypes among patients with ATTR-CM, from HFpEF without LV dilatation to HFrEF with severe LV dilatation. Relying solely on HFpEF for screening may lead to under-diagnosis. These findings suggest the need for more comprehensive diagnostic criteria beyond echocardiographic measures to improve ATTR-CM detection and management.

## Introduction

Transthyretin amyloid cardiomyopathy (ATTR-CM), once considered a rare disease, has seen a significant rise in diagnosis over the years [1,2]. Nevertheless, ATTR-CM is still frequently overlooked due to unawareness of the disease beyond specialized centres, heterogenic and non-specific symptoms, and overlapping conditions with subsequent misdiagnosis [2–4].

Guidelines recommend suspecting ATTR-CM in patients above 65 years of age presenting with unexplained left ventricular (LV) hypertrophy in a non-dilated LV, HF and preserved ejection fraction (HFpEF), hypertrophic cardiomyopathy, or severe aortic stenosis [1,5]. Currently, there is a strong emphasis on HFpEF, whereas there is evidence indicating a high prevalence of ATTR-CM in other HF phenotypes [5,6]. As such, this study aimed to characterize the diversity of HF phenotypes of ATTR-CM by examining the left ventricular (LV) ejection fraction and LV dilatation using echocardiography.

## Methods

This multicentre retrospective observational study included all patients diagnosed with ATTR-CM between 2015–2023 at the University Medical Centre Utrecht (UMCU) [7] and the Maastricht University Medical Centre (MUMC). This study adhered to the Principles of the Declaration of Helsinki and was approved by ethics committees of MUMC and UMCU (non-WMO Amyloid 2022-3440 and UCC-UNRAVEL #12-387). Patients included from the UMCU provided written informed consent. The ethics committee of the MUMC waived the requirement for informed consent for patients included in the MUMC.

The diagnosis of ATTR-CM followed current guidelines [1]. Briefly, a positive cardiac biopsy for TTR amyloid or positive bone scintigraphy (99mTc-HMDP; Perugini grade ≥2) was performed in the absence of monoclonal gammopathy. Bone scintigraphy was followed by single-photon emission computed tomography with low-dose non-contrast computed tomography to rule out false-positive results.

Clinical information, echocardiography findings, and laboratory parameters used for routine clinical care were collected at diagnosis. Echocardiography was performed by certified individuals, and cut-offs were based on the 2023 cardiomyopathy guidelines and recommendations for cardiac chamber quantification [8]. Descriptive analyses were conducted between groups based on LV ejection fraction (LVEF): HFpEF, LVEF ≥50%; HF with mildly reduced EF (HFmrEF), LVEF 40–49%; and HF with reduced EF (HFrEF), LVEF <40%. LV cavity size was subdivided into three groups according to LV end-diastolic diameter (LVEDD) and LV end-diastolic volume index (LVEDVi): normal, moderately increased and severe. For males, the normal LV dimension was defined as LVEDD ≤ 50 mm or LVEDVi ≤54mL/m^2^, while for females it was LVEDD ≤ 45 mm or LVEDVi ≤45mL/m^2^. Moderate increased LV dimensions were considered when LVEDD was 51–58 mm or LVEDVi 55–74mL/m^2^ for males, and LVEDD 46–52 mm or LVEDVi 46–61mL/m^2^ for females. Severe LV dimensions were defined as LVEDD >58 mm or LVEDVi ≥75mL/m^2^ in males and LVEDD >52 mm or LVEDVi ≥62mL/m^2^ in females. Troponins were assessed as troponin I or high-sensitivity troponin T. Elevated troponin levels were defined as levels above the upper reference limit for both troponins. The severity of ATTR-CM at diagnosis was subdivided according to the Gillmore staging criteria [9]. Data were analysed using RStudio version 2021.09.01 (Boston, MA, USA). Statistical significance was set at *p* < 0.05.

## Results

This study included 135 ATTR-CM patients (mean age, 78 years; 89% male; 89% wild-type ATTR-CM; Table 1). Most patients were screened for ATTR-CM owing to the presence of unexplained HF and increased LV wall thickness (57%). Other reasons included unexplained HF combined with extracardiac signs and symptoms of TTR amyloidosis (32%), family screening (3%) and incidental findings (4%).

**Table 1.** Characteristics of ATTR-CM patients with different heart failure phenotypes on echocardiography at time of diagnosis.

Characteristic	Overall	HFrEF	HFmrEF	HFpEF	*p*-value
	*n* = 135	*n* = 42 (31%)	*n* = 39 (29%)	*n* = 54 (40%)	
Male sex	120 (89%)	40 (95%)	35 (90%)	45 (83%)	0.180
Age (years)	78 (± 8)	80 (± 5)	77 (± 9)	77 (± 8)	0.092
**ATTR type**					0.020
ATTRwt	120 (89%)	42 (100%)	32 (82%)	46 (85%)	
ATTRv	15 (11%)	0 (0%)	7 (18%)	8 (15%)	
**NYHA class**					0.086
Class I	16 (12%)	3 (7%)	2 (5%)	11 (20%)	
Class II	83 (62%)	27 (64%)	25 (64%)	31 (57%)	
Class III	29 (22%)	9 (21%)	10 (26%)	10 (19%)	
Class IV	3 (2%)	2 (5%)	1 (3%)	0 (0%)	
**Medical history**					
Hypertension	60 (44%)	22 (52%)	17 (44%)	21 (39%)	0.419
Coronary artery disease	46 (34%)	17 (41%)	14 (36%)	15 (28%)	0.437
Aortic valve stenosis	13 (10%)	5 (12%)	5 (13%)	3 (6%)	0.404
Supraventricular arrhythmia	79 (59%)	25 (60%)	25 (64%)	29 (54%)	0.508
High grade atrioventricular block	17 (13%)	8 (19%)	4 (10%)	5 (9%)	0.322
Carpal tunnel syndrome	65 (48%)	21 (50%)	16 (41%)	28 (52%)	0.637
Extra cardiac amyloidosis	77 (57%)	29 (69%)	16 (41%)	32 (59%)	0.049
**Echocardiography:**					
LVEF (%)	46 (± 11)	34 (± 5)	45 (± 3)	57 (± 6)	< 0.001
IVSd (mm)	16 (±4)	16 (± 4)	17 (± 4)	15 (± 3)	0.156
LVMI (g/m²)	142 (± 42)	162 (± 41)	151 (± 39)	127 (± 39)	0.001
LAVI (ml/m²)	50 (± 16)	52 (± 16)	53 (± 14)	48 (± 18)	0.318
LVEDD (mm)	46 (± 7)	49 (± 7)	46 (± 6)	43 (± 6)	< 0.001
EDVi (ml/m2)	58 (± 10)	61 (± 21)	61 (± 18)	52 (± 20)	0.165
E/e’ average	14.4 (± 6.4)	15.3 (± 6.5)	13.4 (± 5.5)	14.5 (± 6.9)	0.494
LV cavity dimension group					0.014
Normal	67 (50%)	13 (31%)	20 (51%)	34 (63%)	
Moderate dilatation	41 (30%)	18 (43%)	12 (31%)	11 (20%)	
Severe dilatation	18 (13%)	7 (17%)	6 (15%)	5 (9%)	
**Laboratory:**					
eGFR (ml/min/1.73m²)	61.9 (± 18.3)	56.6 (± 16.3)	63.5 (± 16.6)	64.9 (± 21.0)	0.083
NTproBNP (pg/ml)	2429 [1123 − 4083]	2795 [2048 − 4831]	3391 [1666 − 4110]	1664 [809 – 2985]	0.005
Elevated troponin	74 (55%)	24 (57%)	27 (69%)	23 (43%)	0.010
**Gilmore prognostic** score					0.158
Score I	66 (49%)	18 (43%)	18 (46%)	31 (57%)	
Score IIa	10 (7%)	3 (7%)	1 (3%)	6 (11%)	
Score IIb	35 (26%)	11 (26%)	16 (41%)	8 (15%)	
Score III	12 (9%)	5 (12%)	3 (8%)	4 (7%)	

Data are presented as *n* (%), mean ± standard deviation, or median [interquartile range].

**Gillmore prognostic score:**

**STAGE I**, NT-proBNP ≤ 3000 pg/ml & eGFR ≥ 45 ml/min;.

**STAGE IIa**, NT-proBNP ≤ 3000 pg/ml & eGFR < 45 ml/min;.

**STAGE IIb**, NT-proBNP > 3000 pg/ml & eGFR ≥ 45 ml/min;.

**STAGE III**, NT-proBNP > 3000 pg/ml & eGFR < 45 ml/min.

**Left ventricle cavity dimension groups:**

**Normal**: Males: LVEDD ≤ 50 mm or LVEDVi ≤54mL/m^2^; Females LVEDD ≤ 45 mm or LVEDVi ≤45mL/m^2^.

**Moderate dilatation**: Males: LVEDD was 58 mm; LVEDVi 55-74mL/m^2^; Females: LVEDD 46–52 mm or LVEDVi 46-61mL/m^2^.

**Severe dilatation**: male: LVEDD >58 mm or LVEDVi ≥75mL/m^2^; female: LVEDD >52 mm or LVEDVi ≥62mL/m^2^.

Abbreviations: ATTR: transthyretin amyloidosis; ATTRwt: wild-type transthyretin amyloidosis; ATTRv: variant transthyretin amyloidosis; NYHA: New York Heart Association; LVEF: left ventricular ejection fraction; IVSd: intraventricular septum diameter; LVMI: left ventricular mass index; LAVI: left atrial volume index; LVEDD: left ventricular end diastolic diameter; EDVi: end diastolic volume index; LV: left ventricular; eGFR: estimated glomerular filtration rate; NTproBNP: N-terminal pro-B-type natriuretic peptide.

Echocardiography revealed an LVEF of <50% in 81 (60%) patients, with the majority (*n* = 43) presenting with HFrEF (Table 1). Patients with LVEF <50% were more likely to present with a higher NYHA class compared to those with HFpEF (*p* = 0.028). Patients with HFrEF or HFmrEF demonstrated significantly higher levels of N-terminal pro-B-type natriuretic peptide (2795 pg/ml and 3391 pg/ml *vs.* 1664 pg/ml respectively; *p* = 0.005), and more patients with HFrEF or HFmrEF had elevated troponin levels (57% and 69% *vs*. 43%, respectively; *p* = 0.010) than those with HFpEF (Table 1). Consequently, a higher but not significant proportion of patients with HFrEF or HFmrEF were observed in the more advanced prognostic stages II and III than in patients with HFpEF (45% and 51% *vs*. 33%, respectively; *p* = 0.247). There was no significant difference in the time period in which the ATTR-CM diagnosis was made (T1 2015–2020; T2 2021–2023), between patients with LVEF <50% and those with LVEF ≥50 (T1:30% LVEF < 50% and 39% LVEF ≥ 50% *vs*. T2 70% LVEF < 50% and 61% LVEF ≥ 50%; *p* = 0.351).

**Figure 1.**
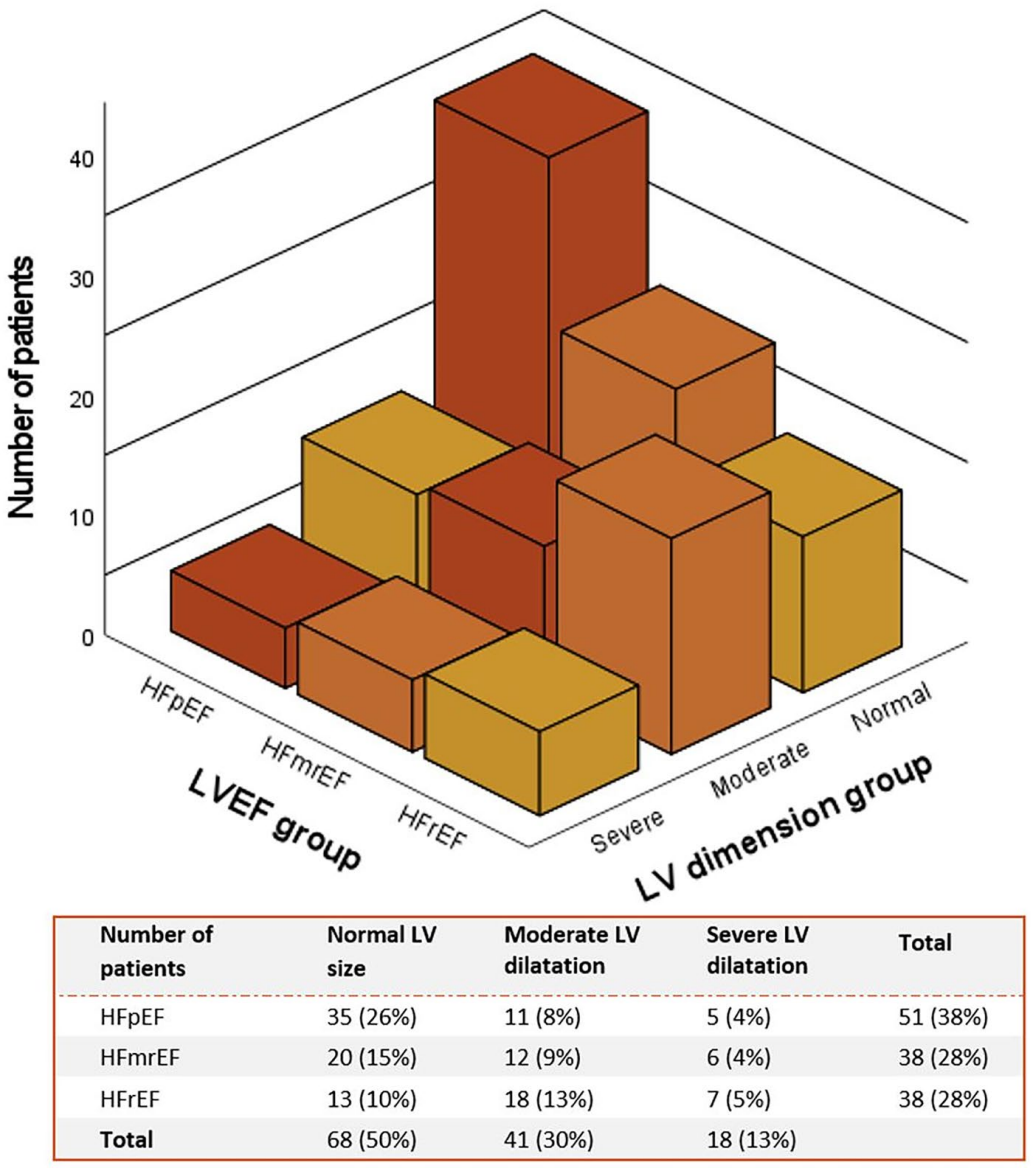
Distribution of patients presenting with different heart failure phenotypes and different patterns of left ventricular dimension in transthyretin cardiac amyloidosis. LV dimension groups: Normal LV size: Male: LVEDD ≤ 50 mm or LVEDVi ≤54mL/m^2^; female: LVEDD ≤ 45 mm or LVEDVi ≤45mL/m^2^. Moderate LV dilatation: Males: LVEDD was 58 mm; LVEDVi 55-74mL/m^2^; female: LVEDD 46–52 mm or LVEDVi 46-61mL/m^2^. Severe LV dilatation: male: LVEDD >58 mm or LVEDVi ≥75mL/m^2^; female: LVEDD >52 mm or LVEDVi ≥62mL/m^2^. Abbreviations: HFrEF: heart failure with reduced ejection fraction <40%; HFmrEF: heart failure with mildly reduced ejection between 40% and 50%; HFpEF: heart failure with preserved ejection fraction ≥ 50%; LVEF: left ventricular ejection fraction; LV: left ventricular; LVEDD: left ventricular end-diastolic diameter; LVEDVI: left ventricular end-diastolic volume index.

Regarding LV dimensions, moderate LV dilatation was observed in 41 (30%) patients and severe LV dilatation was observed in 18 (13%) ATTR-CM patients. As depicted in Figure 1, the HF presentation of ATTR-CM patients varied widely from HFpEF with normal LV dimensions (*n* = 34, 25%) to HFrEF with severe LV dilatation (*n* = 7, 5%). Thirteen patients (10%) presented with LVEF < 50% and severe LV dilatation, which corresponded to the definition of dilated cardiomyopathy. Interestingly, ATTR-CM patients with LVEF < 50% and severe LV dilatation were not present in the more advanced prognostic Gillmore stages II and III compared with the other HF phenotypes (31% *vs*. 47%, *p* = 0.365).

Finally, we observed no significant differences between men and women with ATTR-CM based on LVEF class (*p* = 0.240) and LV dilatation class (*p* = 0.313), despite the relatively low number of female patients in our study cohort.

## Discussion

In the light of the screening recommendations of the European Society of Cardiology (ESC) [1], our study has several interesting findings. First, the majority of ATTR-CM patients (60%) presented with LVEF less than 50% and showed signs of more advanced disease. Second, 43% of ATTR-CM patients presented with LV dilatation and even one in 10 ATTR-CM patients presented with LVEF < 50% and severe LV dilatation. Concluding that only 26% of our cohort would have been screened for ATTR-CM if we strictly adhered to the ESC guidelines.

Our finding that ATTR-CM patients present with (mildly) reduced LVEF aligns with previous findings [10,11]. The large international Transthyretin Amyloidosis Outcomes Survey (THAOS) registry reports a median LVEF of 55% [46-66] in wild-type ATTR-CM patients [12]. Additionally, it has been shown that screening for ATTR-CM in HFmrEF and HFrEF patients without a known aetiology, results in a 10% detection rate [13,14]. This led to a recent consensus paper from the World Heart Federation, which supports that a proportion of ATTR-CM patients have an LVEF <50% [5]. However, our data indicate that an LVEF < 50% is even more prevalent than HFpEF in ATTR-CM patients. Nevertheless, the new consensus document emphasizes the importance of considering ATTR-CM in HF patients without an identified cause across the full spectrum of LVEF values [5].

In addition to (mildly) reduced LVEF, we observed LV dilation in a significant portion of our patients. This is noteworthy, as both the ESC position statement and World Heart Federation consensus paper recommend considering ATTR-CM in cases with a non-dilated LV [1,5]. To our knowledge, this finding has not been previously described. The THAOS registry also reports no significant dilation, with a median LVEDD of 45 mm (range 42-50 mm). However, there is a published case report describing a patient with dilated cardiomyopathy who was diagnosed with ATTR-CM after heart transplantation [15].

Lastly, our study demonstrates that patients with (mildly) reduced LVEF presented with more advanced disease. Previous studies have shown that these patients also have a poorer prognosis [16,17]. Whether ATTR-CM with LVEF < 50% and/or LV dilatation represents the natural progression of ATTR-CM to a ‘burn out’ phase of cardiomyopathy [11] or represents a different entity needs to be determined through long-term follow-up studies in ATTR-CM patients. Nevertheless, we found no significant differences in comorbidities between patients with LVEF <50% and those with HFpEF. This contradicts the notion that the occurrence of LVEF <50% in these patients results from a combination of factors and/or comorbidities. Furthermore, it is known that after 24 months, LVEF declines, particularly in hereditary patients with the V112Ile mutation, while the LV cavity size decreases [18]. Diagnosing patients at an early stage remains challenging [2,19], and our study shows that the proportion of HFpEF and HFrEF/HFmrEF patients identified in recent years has remained comparable. With emerging treatment options capable of resolving TTR amyloid deposits, it is crucial to identify ATTR-CM patients, even in advanced disease stages [20]. However, future research is necessary to determine whether positive bone scintigraphy in patients with other HF phenotypes can be considered indicative of amyloid-related disease or whether it is an incidental subclinical finding. This is particularly relevant given that the prevalence of ATTR-CM increases with age [21]. The question remains whether all of these patients had HF directly attributable to ATTR-CM, or if ATTR-CM can be present without necessarily leading to overt HF.

Our findings could be influenced by selection and referral biases, potentially leading to a higher-than-anticipated proportion of patients with HFrEF and LV dilatation.

## Conclusion

In conclusion, this study demonstrated considerable variability in the HF phenotype of ATTR-CM, ranging from HFpEF without LV dilatation to HFrEF with severe LV dilatation. Exclusively relying on HFpEF as the first screening parameter for the diagnosis of ATTR-CM would have resulted in the underdiagnosis of the majority of ATTR-CM patients. These findings highlight the potential shortcomings of the current diagnostic guidelines, necessitating the development of more specific indicators for ATTR-CM detection beyond echocardiographic measures.

## Data Availability

The data that support the findings of this study are available upon request from the corresponding author, AA. The data were not publicly available because of privacy restrictions.
